# Strengthening Social and Behavior Change in Postabortion Care: A Call to Action for Health Professionals

**DOI:** 10.9745/GHSP-D-18-00307

**Published:** 2019-08-22

**Authors:** Erin Mielke, Hope Hempstone, Ashlie Williams

**Affiliations:** a Bureau for Global Health, U.S. Agency for International Development, Washington, DC, USA.

## Abstract

Social and behavior change approaches have shown promise for addressing the demand- and supply-side challenges in postabortion care. As implementers seek to improve the quality of postabortion care, systematically integrating long-standing models and emerging approaches, including behavioral economics, human-centered design, and attribute-based models of behavior change, can promote positive health outcomes.

## INTRODUCTION

Unsafe abortion is the fifth leading direct cause of maternal deaths globally and in developing countries.^1^ Postabortion care (PAC) programs, encompassing both facility-based and community-based interventions, are essential to address complications of induced and spontaneous abortion, prevent subsequent unintended pregnancies, and ensure healthy timing and spacing of intended pregnancies. The 3 key components of PAC programs are (1) emergency treatment for complications of spontaneous or induced abortion, (2) family planning counseling and service provision, and (3) community engagement.^2^ However, access to and provision of high-quality PAC are compromised in many settings by pervasive social stigma around induced and spontaneous abortion, provider bias, and complex counseling requirements. These barriers impede timely care seeking for complications of abortion and limit access to the full range of voluntary family planning methods during PAC. Social and behavior change, which is widely recognized to be a key component of effective reproductive health programming, offers particular potential to improve the quality and impact of PAC. Social and behavior change approaches include outreach and community mobilization, counseling and interpersonal communication, behavioral “nudges,” and use of digital platforms targeting clients or providers, among many others. Robust application of these approaches can motivate health-seeking behaviors before, during, and after the time a client enters a health facility for PAC and shape the care a client receives in a facility. Achieving positive outcomes among PAC clients depends on the extent to which programs (1) attend to the varied needs of different client groups and (2) support effective interaction of providers and clients in high-quality counseling and follow-up care. Both factors are key tenets of effective behavior change programming. PAC programs that successfully conduct client-centered demand creation, counseling, and follow-up and address the full range of barriers to delivery of high-quality and nonjudgmental care will be well equipped to overcome the common challenges in PAC programming discussed in this article.

## ADDRESSING COMMON PAC CHALLENGES THROUGH SOCIAL AND BEHAVIOR CHANGE

Programmatic experience suggests that key behavior-related challenges in the delivery of high-quality PAC services arise before the PAC client reaches a facility, during her visit to a facility, and after she leaves a facility. These challenges include the following^3^:

**Before:**

- Ensuring timely care seeking by women and their partners

**During:**

- Promoting respectful treatment of PAC clients by providers and addressing the stigma that specific client groups may experience in interactions with providers
- Ensuring that during counseling, providers address client needs for clear information about return to fertility and family planning options and acknowledge barriers to postabortion family planning
- Ensuring providers offer a full range of voluntary family planning methods prior to discharging the client, including long-acting reversible contraceptives and permanent methods

**After:**

- Supporting clients in returning to services to facilitate both voluntary contraceptive continuation and follow-up for clients whose treatment requires return visits

Key challenges in the delivery of high-quality PAC services arise before, during, and after a PAC client’s visit to a facility.

While additional formal evaluation of social and behavior change in the context of PAC is needed, lessons learned from programmatic experience in this and other areas of reproductive health suggest that intentional use of behavior change principles and approaches is necessary to address common challenges in PAC programming.

### Before PAC Clients Reach a Facility

Seeking care at the first sign of complication is essential to effective treatment of spontaneous and induced abortions. Facilitating timely care seeking requires attention to barriers to accessing services, which may be specific to a given context or client group. Lack of information about the warning signs of spontaneous abortion, dangerous complications of spontaneous and induced abortion, and where to seek PAC prevents women from seeking help.^4^^,^^5^ In many settings, fear of abuse, ill treatment, or legal reprisal arising from the stigma associated with abortion also deters women from seeking medical care.^5^ Youth face particular barriers to timely care seeking, including lack of trust in providers, concerns about confidentiality, and fear of being stigmatized for engaging in risky behaviors.^6^^,^^7^

PAC programs have successfully intervened at the community level to address barriers to care seeking and reinforce supportive norms. In Kenya and Somalia, community education, training of community health workers and community preparedness have effectively increased awareness, acceptance, and utilization of PAC, including voluntary use of postabortion family planning.^8^^–^^10^ Organizations in Bolivia and Kenya successfully applied the community action cycle to learn about the concerns of distinct segments of the local population (e.g., women, men, and adolescents). In turn, community members learned about PAC services, participated in ensuring service delivery, and demonstrated ownership of programs to improve access to and use of PAC.^11^^–^^13^ In Kenya, this approach reduced stigma among family and community members and improved their care-seeking responses to bleeding during pregnancy.^11^

PAC programs have successfully intervened to address community-level barriers to care seeking and reinforce supportive norms.

PAC programs must consistently apply proven practices in behavioral design, including systematic diagnosis of barriers to service utilization among key client groups. In addition to addressing the common barriers cited above, implementers should employ formative research approaches used in behavioral programming as a means to generate insights about client perceptions and motivations. In particular, immersive and participatory approaches grounded in human-centered design show promise as a means to better understand and address barriers to care seeking. Human-centered design is an iterative process that includes developing a deep understanding of the people affected by a problem, identifying opportunities for design to address that problem, prototyping potential solutions, and repeatedly testing and refining those prototypes.^14^ The end-user is directly engaged throughout each stage of this process. In Côte d’Ivoire, for example, a human-centered design project employed group interviews, field observations, and participatory brainstorming and prototyping workshops with stakeholders to understand barriers to family planning use among young men working in the informal sector and their partners.^15^ A deep understanding of barriers can inform both the development of social and behavior change messages and the selection of communication channels or approaches.^16^ Postabortion care seeking may also be improved by promoting utilization of reproductive health services as an entire category through proven approaches such as branding of services, mass media campaigns, community activation events, or targeted interpersonal communication. With regard to facilitating youth access to reproductive health services, multiple studies show that promotion of reproductive health care via school and community outreach and mass media improves care seeking among young people and positively influences parental and other gatekeepers’ approval of reproductive health services for youth.^17^^–^^19^ This improvement is attained by increasing awareness of services and trust in providers and the health system as an entity *before* emergency care is required. 

### During a PAC Client’s Visit to a Health Facility

The client-provider interaction during health care provision represents a critical opportunity to build trust and establish healthy behaviors pertaining to family planning and reproductive health care seeking. Four distinct but interrelated challenges may influence the quality of care offered during a PAC client’s visit to a health facility. Failing to prevent these challenges can ultimately hinder adoption of voluntary family planning methods that enable women and their partners to achieve their desired fertility. First, **providers may treat clients with disrespect** or restrict care due to norms or attitudes associated with abortion, sexuality, and sexual health among specific client groups; circumstances such as a subsequent abortion or failure to use family planning; and/or use of family planning after pregnancy loss.^7^^,^^20^^–^^22^ Second, **providers may not adequately explain healthy spacing of pregnancy.** The World Health Organization recommends women delay pregnancy for at least 6 months after an abortion^23^ (although a recent meta-analysis in mainly developed countries showed a shorter interval following miscarriage is not associated with adverse outcomes^24^). Similarly, **providers may not adequately explain return to fertility**, which can occur within 2 weeks after a first trimester spontaneous or induced abortion.^23^ One cross-sectional study in Egypt showed that two-thirds of 412 women who received PAC did not know when fertility returns after abortion and were not intending to use contraception, despite three-quarters wanting to postpone childbearing.^25^ Women in Russia who left the health facility without a clear understanding of their return to fertility following abortion were 4 times more likely to have a repeat abortion than those with accurate knowledge.^26^ Finally, **providers may fail to counsel PAC clients on the full range of family planning methods available to them** and fail to offer these methods prior to discharging the client.^26^^,^^27^ Incomplete counseling and failure to offer voluntary family planning may be influenced in part by assumptions about client preferences, provider bias toward certain family planning methods (which providers perceive to be safer following an abortion or more appropriate for certain client groups), or low motivation among providers and lack of performance feedback.^28^^–^^29^

To prevent these challenges, PAC programming has successfully employed a range of approaches to influence provider behaviors pertaining to counseling PAC clients, particularly about postabortion family planning. Many PAC programs have integrated discussions of provider attitudes and values into trainings that seek to reduce stigma toward women and adolescents who need reproductive health services, dispel misconceptions about contraceptive methods, and build effective counseling skills. These discussions offer opportunities for providers to reflect on how their own values and beliefs influence their interactions with clients.^30^^,^^31^ Adaptable training materials to build empathy with different types of clients and distinguish between personal beliefs and professional responsibilities are available.^32^ Similarly, attention to expanding the contraceptive method mix offered to PAC clients has increased voluntary uptake of highly effective methods. Programs in Ethiopia and Guinea enhanced access to long-acting reversible contraceptives for PAC clients and noted that a broader method mix can satisfy clients with a variety of needs.^33^^,^^34^ To support sustained improved practice, some Latin American PAC programs incorporated complementary provider behavior change approaches, such as the use of peer opinion leaders to promote best practices and strategic placement of visual reminders of clinical guidelines.^35^ Across 10 countries in Asia and sub-Saharan Africa, provider behavior change was reinforced by post-learning follow-up by mentors to reinforce new skills, provide performance feedback, and resolve obstacles to care.^32^ Provider job aids that improve motivation and enable facilities to track performance improvements have proved effective in Ethiopia.^36^^,^^37^ Finally, recent years have seen growing use of behavioral economics (i.e., applying psychological insights such as non-conscious biases and mental models that influence decision making) to promote provider behavior change. In Nepal, for example, one intervention made effective use of peer comparison approaches to allow providers to compare their health center’s performance to other similar centers, increasing providers’ motivation to improve quality of counseling and increasing voluntary family planning uptake during PAC.^29^

PAC programming has successfully employed several approaches to influence provider behaviors in counseling PAC clients.

PAC programs can build on the rapidly growing evidence base for provider behavior change as a means to improve not only the knowledge and skills that underpin effective counseling, but the full range of motivational and normative influences affecting providers. To do so, implementers must use formative research to analyze specific drivers of provider behavior. As with programs targeting PAC clients themselves, the insights derived from this research can inform segmentation^38^ and profiling of providers and identify important behavioral determinants, thus better targeting interventions to change provider behavior. Provider behavior change approaches must also clearly define specific, measurable behaviors and practices that constitute quality PAC counseling and respectful care, such as engagement with clients as active participants in a 2-way counseling dialogue. Adherence to these and other proven practices in behavioral design offer potential to deepen the impacts of more traditional knowledge- and skills-based interventions for improving client-provider interaction.

Behavioral design may deepen the impact of traditional knowledge- and skills-based interventions to improve client-provider interaction.

PAC programs can further improve the quality of client-provider interaction through continued attention to the drivers of client behavior, specificity in behavior change messaging, and promotion of client engagement during care and counseling. Counseling and other communication approaches should acknowledge and address barriers to postabortion family planning, including social norms regarding fertility and couples’ communication.^39^ Activities that improve the quality of counseling can affect not only uptake of family planning but also its continuation.^40^ In Northeast Brazil, for example, female PAC clients who received counseling personalized to their plans for future contraceptive use and their beliefs and previous experiences were 41% more likely to be using a contraceptive method 6 months after an abortion than those who received only standardized information.^41^ In addition, activities that directly empower clients to pose questions and express their reproductive intentions can prompt providers to offer further information and address clients’ concerns, as demonstrated by a community education and mass media program in Indonesia.^42^ Such approaches may be particularly relevant for young PAC clients who wish to delay or space childbearing but are not using contraception.

### After a PAC Client Leaves a Facility

PAC programming must address challenges relating to support for voluntary contraceptive initiation or continuation and care for clients who require follow-up treatment. Clients who receive counseling and choose a voluntary family planning method may require assistance with accessing ongoing supplies of short-acting methods^43^ or follow-up to obtain long-acting or permanent methods when it is not possible to obtain these during the initial visit or referral to a different facility is required.^5^ Women who choose an intrauterine device or tubal ligation following treatment with misoprostol, for example, require a follow-up visit to ensure that evacuation of the uterus is complete and no complications are present, such as prolonged heavy bleeding or fever.^5^

Social and behavior change interventions within PAC programs enhance a client’s follow-up care, including family planning, and reinforce linkages between the client’s home and health care workers. Client communication materials facilitate follow-up care. Several international professional associations and development partners recommend that PAC clients should receive simple written instructions for the use of their method, along with concise information about common side effects and benefits.^44^ In Sri Lanka, women who received an informational leaflet during antenatal care were significantly more likely to accept an intrauterine device following delivery.^45^ Providing take-home information during PAC may similarly support self-care and adoption or continuation of family planning after treatment for complications of a spontaneous or induced abortion. Examples of such client communication materials and provider training materials can be found online and may be adapted to the needs of specific audiences or cultural contexts as needed.^46^^,^^47^

Social and behavior change interventions within PAC programs enhance a client’s follow-up care.

Mobile phone-based communication has shown promise to facilitate access to health information and services in low-resource settings. A randomized control trial in Cambodia found that follow-up communication through mobile phones supports postabortion contraceptive use. It helped women retain family planning information provided during PAC and prompted women who wanted a long-acting method but did not receive one during the initial PAC visit to return for an implant or intrauterine device.^48^ This channel of communication was as effective as home visits in sustaining use of long-acting methods at 12-month follow-up post insertion in rural Punjab, Pakistan.^49^ Mobile phones are an effective tool for facilitating access to care and information and promoting behavior change among youth.^50^ PAC programs considering mobile phone–based communication must give careful attention to risks to confidentiality, such as shared use of a mobile phone among family members.

Evidence suggests that engaging the male partners of PAC clients in counseling, when acceptable to clients themselves, can improve health outcomes. PAC programs in Bolivia and Tanzania found that engaging partners by informing them of the client’s condition and providing both with family planning counseling helped to ensure safe recovery and increased voluntary family planning continuation.^51^^,^^52^ In China and Egypt, counseling partners on follow-up care, postabortion warning signs, sources of referral care, return to fertility, and family planning also increased family planning usage and physical, material, and emotional support for the clients during recovery.^53^^,^^54^

**In general, promotion of voluntary contraceptive continuation or return to services remains less understood than either creation of initial demand for services or improvement of client-provider interaction.** Improved design and measurement of activities addressing behavioral initiation or maintenance after a client leaves a facility is an area of increasing collaboration between social and behavior change and service delivery professionals. The concerns of PAC program implementers constitute an important component of this discourse. The rapidly evolving thinking in this area affords PAC program implementers an opportunity to build upon emerging practices and develop innovative solutions to the challenges they face. For example, a growing body of gray literature pertaining to understanding and grouping similar behaviors and the attributes of those behaviors^55^^,^^56^ may allow for application of lessons learned from the promotion of other private, stigmatized, or provider-dependent behaviors. Interventions informed by behavioral economics to facilitate health seeking after PAC merit exploration given the amount of information associated with postabortion recovery, return to fertility, and adoption or continuation of family planning and with the normative and emotional context in which clients consider this information. Finally, reproductive “self-care” and direct-to-consumer family planning methods (i.e., not requiring a health worker to administer, such as condoms, oral contraceptives, or mobile phone applications to track fertile days once a woman’s regular menstrual cycle resumes), which are increasingly facilitated by mobile technology, may be particularly relevant in the context of PAC programming.

We should build our understanding of how to address behavioral initiation or maintenance after a client leaves a facility.

## CONCLUSION

PAC programs have successfully used community-based social and behavior change approaches and provider behavior change interventions to support clients in accessing postabortion services and adopting voluntary family planning. To ensure that all women have access to high-quality PAC, implementers must build upon this important foundation through continued application of proven practices in behavior change, including formative research, audience segmentation, and the use of iterative, multichannel communication approaches that target clients, providers, and community members. Concomitantly, implementers should seize the opportunity to play a leading role in the application of social and behavior change approaches for PAC by leveraging new trends in behavioral economics, human-centered design, and attribute-based models of behavior change and increasing alignment between demand-side and supply-side programming.

## Notes

**Peer Reviewed**

**Funding:** This work received no specific grant from any funding agency in the public, commercial, or not-for-profit sectors.
